# Multi-omics atlas of ovarian cellular and molecular responses to diabetes

**DOI:** 10.1016/j.molmet.2025.102307

**Published:** 2025-12-13

**Authors:** Zheng-Hui Zhao, Xue-Ying Chen, Cheng-Yan Zhuo, Xiang-Hong Ou, Qing-Yuan Sun

**Affiliations:** Guangzhou Key Laboratory of Metabolic Diseases and Reproductive Health, Guangdong-Hong Kong Metabolism & Reproduction Joint Laboratory, Reproductive Medicine Center, the Affiliated Guangdong Second Provincial General Hospital of Jinan University, Guangzhou, 510317, China

**Keywords:** Diabetes, Ovary, Single-cell RNA sequencing, DNA methylation, Metabolism

## Abstract

Diabetes is associated with compromised reproductive health; however, the cellular and molecular mechanisms underlying its impact on ovarian function remain largely unclear. In this study, we integrated single-cell RNA sequencing, DNA methylation profiling, and metabolomic analyses to comprehensively characterize the ovarian cellular landscape, epigenetic alterations, and metabolic reprogramming in diabetic female mice, with a focus on identifying diabetes-induced changes in ovarian cells. Our cell type-specific transcriptomic analysis revealed that dysregulated steroid hormone biosynthesis and impaired fatty acid metabolism are prominent features of diabetic ovarian dysfunction. Notably, key genes including *Cyp11a1*, *Fshr*, and *Lhcgr* exhibited reduced expression accompanied by increased DNA methylation levels in their gene regions within granulosa cells under diabetic conditions. Furthermore, disrupted granulosa cell differentiation was evident, leading to aberrant luteal cell formation and compromised luteal function. In parallel, metabolomic profiling revealed profound metabolic reprogramming in diabetic ovaries, with significant alterations in lipid metabolism pathways, including elevated unsaturated fatty acid and reduced glycerophospholipid metabolism. Taken together, these findings provide novel insights into the molecular pathways underlying ovarian dysfunction in the context of diabetes, thereby enhancing our understanding of folliculogenesis in metabolic disorders.

## Highlights

• Diabetes impairs ovarian steroidogenesis and fatty acid metabolism via Cyp11a1 and Lhcgr downregulation.
• Increased DNA methylation is observed at the *Cyp11a1* and *Lhcgr* gene loci in diabetic ovaries.
• Diabetic conditions disrupt granulosa-to-luteal cell differentiation.
• Metabolomic profiling reveals marked reprogramming of lipid metabolism in diabetic ovaries.

## 1 Introduction

Type 1 diabetes mellitus (T1DM), is a chronic metabolic disorder characterized by insulin deficiency and persistent hyperglycemia. The dysregulation of glucose metabolism is a major contributor to reproductive impairment at different levels of the gonadotropic axis [1]. Several studies have demonstrated an association between decreased luteinizing hormone (LH) response to gonadotropin-releasing hormone (GnRH) stimulation and increased fasting glucose levels [2,3]. Moreover, women with poorly controlled T1DM exhibit features of hypogonadotropic hypogonadism, characterized by low circulating levels of LH, follicle-stimulating hormone (FSH), and estradiol [4,5]. Furthermore, the pituitary in T1DM patients demonstrates a normal response to exogenous GnRH administration, indicating that the hypogonadism is centrally mediated and likely attributable to hypothalamic dysfunction [6,7]. Similar to human studies, the rodent models of uncontrolled diabetes also exhibit a marked hypogonadotropic state, characterized by reduced basal levels of gonadotrophins and sex steroids, reflecting disrupted endocrine regulation within the hypothalamic-pituitary-gonadal axis [8,9]. Moreover, female diabetic models display impaired estradiol-induced positive feedback, characterized by delayed or absent preovulatory LH peaks and anovulatory cycles [10].

As a central component of the hypothalamic-pituitary-ovarian axis, the ovary integrates neuroendocrine signals and provides feedback regulation via steroid hormone production to maintain reproductive homeostasis. Emerging evidence indicates that ovarian dysfunction contributes significantly to the reproductive impairments associated with T1DM. For instance, diabetic female mice displayed reduced numbers of follicles, increased apoptosis in granulosa cells, impairment of oocyte-to-granulosa communication, delayed oocyte maturation, and significantly decreased ovulation rates [[11], [12], [13]]. Moreover, diabetes impacts offspring through its effects on oocytes and the intrauterine environment during pregnancy, resulting in embryonic developmental defects, miscarriage, preterm birth, fetal malformations, and metabolic changes in offspring [14,15]. Therefore, identifying target cells and elucidating the molecular mechanisms by which diabetes influences ovarian function are crucial for maintaining and preserving female fertility and mitigating the intergenerational impact of diabetes.

Recent advances in high-throughput omics technologies have provided powerful tools to dissect the complex interplay between metabolic dysregulation and reproductive dysfunction. While single-cell RNA sequencing (scRNA-seq) enables precise mapping of cellular heterogeneity and gene expression dynamics at unprecedented resolution, its integration with epigenomic and metabolomic profiling offers a more comprehensive view of disease mechanisms. In this study, we employ a multi-omics approach combining scRNA-seq, DNA methylation analysis, and untargeted metabolomics to systematically investigate how diabetes perturbs ovarian function at multiple regulatory layers. By capturing transcriptional, epigenetic, and metabolic alterations in parallel, we identify cell-type-specific molecular changes, uncover dysregulated pathways linking systemic metabolism to ovarian physiology, and elucidate potential mechanistic links between hyperglycemia and impaired folliculogenesis. The findings from this study not only provide new insights and theoretical guidance for maintaining and protecting fertility in diabetic women clinically but also provides a valuable framework for identifying novel diagnostic markers and therapeutic targets.

## 2 Methods

### 2.1 Animals

All experimental mice were on a C57BL/6J genetic background. The animals were housed under specific pathogen-free (SPF) conditions at the Animal Center of Guangdong Second Provincial General Hospital, Guangzhou, China. Environmental conditions were strictly controlled with a 12-hour light/dark cycle, ambient temperature maintained between 20 and 22 °C, and relative humidity kept at 50–70%. Standard rodent chow and drinking water were provided ad libitum throughout the study. All animal handling and experimental procedures were performed in accordance with protocols approved by the Institutional Animal Care and Use Committee of Guangdong Second Provincial General Hospital.

### 2.2 Diabetic mouse model

Female C57BL/6 mice at 10 weeks of age were acclimated to the housing facility for 2–3 days prior to experimental initiation. Following acclimation, mice were randomly assigned to one of two treatment groups: T1DC group or T1DM group. Mice in the T1DC group received intraperitoneal injections of vehicle buffer, while those in the T1DM group were administered a single high dose of streptozotocin (STZ; 230 mg/kg body weight) to induce experimental diabetes. Animals were maintained for 30 days post-injection. Fasting blood glucose levels were measured after an overnight fast on day 30. Mice with glucose levels ≥17 mM were considered to have developed diabetes and were included in the T1DM group for subsequent analyses.

### 2.3 Ovary digestion and single cell RNA-seq libraries construction

The ovaries from 3-month-old healthy and diabetic mice were used for scRNA-seq experiment. In brief, four ovaries were collected from four individual mice and dissociated into single-cell suspensions using the Tumor Dissociation Kit (#130-095-929, Miltenyi Biotec) according to the manufacturer's instructions. The resulting cell suspensions were filtered, washed, and resuspended in PBS containing 0.04% BSA at a concentration of 300–600 live cells per microliter. Library preparation was carried out using the Chromium Single-Cell 3′ Library & Gel Bead Kit v3 (10x Genomics) following the manufacturer's protocol. Approximately 8,000 cells per sample were loaded onto the 10x Genomics Chromium Controller to generate gel bead-in-emulsion (GEM) partitions. The estimated number of recovered cells per sample was approximately 6,000. Following cell lysis and mRNA capture, barcoded cDNA was synthesized via reverse transcription and subsequently amplified. Final sequencing libraries were generated after fragmentation, adapter ligation, and size selection, and were sequenced on an Illumina NovaSeq 6000 platform.

Given that mammalian oocytes are too large to be processed using the standard microfluidic-based droplet system of 10x Genomics, we employed Smart-seq2, a full-length transcriptome amplification method, for oocyte transcriptomic profiling [16]. Three individual oocytes were collected from each of the T1DC and T1DM groups. Oocytes were manually isolated and lysed in a low-volume reaction mix containing lysis buffer and RNase inhibitor. Full-length cDNA synthesis was performed via reverse transcription using oligo(dT) primers, followed by PCR amplification. The amplified cDNA was purified and assessed for quality using a Fragment Analyzer (Advanced Analytical) and quantified using a Qubit fluorometer. Sequencing libraries were then prepared and subjected to paired-end sequencing on the Illumina NovaSeq 6000 platform.

### 2.4 Processing of scRNA-seq data

Single-cell RNA sequencing data generated from the Illumina NovaSeq 6000 platform were processed using the Cell Ranger pipeline (v4.0.0; 10x Genomics). Briefly, raw binary base call files were first demultiplexed into FASTQ format using the mkfastq function. Resulting FASTQ files were then aligned to the mouse reference genome (mm10) using the STAR aligner. Gene-barcode count matrices were generated and filtered using default parameters to retain high-quality cells and valid transcripts. The filtered count matrices were imported into R (v4.1.0) for downstream analysis using the Seurat package (v4.1.0) [17]. A Seurat object was constructed by integrating scRNA-seq datasets from both the T1DC and T1DM groups. Quality control was performed to remove low-quality cells based on the following criteria: (1) fewer than 200 detected genes per cell, or (2) greater than 5% mitochondrial gene content. Following normalization using the NormalizeData function, highly variable genes (HVGs) were identified using the FindVariableFeatures method. These HVGs were then used for principal component analysis (PCA), and cell clustering was performed in the PCA-reduced space at an optimized resolution. Dimensional reduction and visualization were further carried out using UMAP [18]. Cluster identities were annotated based on known marker gene expression. Differential gene expression analysis between clusters was performed using the FindAllMarkers function with the Wilcoxon rank-sum test as the statistical method. Genes with |log_2_FoldChange| > 0.25 and a false discovery rate (FDR)-adjusted P value < 0.01 were considered differentially expressed. For the Smart-seq2-based single-oocyte RNA-seq data, FASTQ files were first processed using Trim Galore to remove low-quality reads and adapter sequences. Cleaned reads were then aligned to the mm10 reference genome using HISAT2, and gene-level read counts were quantified using FeatureCounts. The resulting count matrix was analyzed using DESeq2 to identify DEGs between oocytes from the T1DC and T1DM groups.

### 2.5 Gene ontology and gene set enrichment analysis

GO analysis and GSEA were conducted using Metascape and ClusterProfiler [19] respectively to explore the DEGs associated biological processes and pathways.

### 2.6 Analysis of cell–cell communication

Cell–cell communication was inferred from the scRNA-seq data using the CellChat package [20]. The built-in “CellChatDB.mouse” database, which contains curated ligand–receptor interaction pairs, was used to map potential signaling events across cell types. To compare intercellular communication patterns between the T1DC and T1DM groups, CellChat objects from both conditions were merged using the mergeCellChat function. Differential communication strength and interaction frequency among cell types were identified using the compareInteractions function and visualized via the netVisual_diffInteraction plotting tool.

### 2.7 Pseudotime analysis

Pseudotime analysis of granulosa and luteal cells was performed using the Monocle 2 R package (v2.0) to reconstruct putative developmental trajectories [21]. A gene expression count matrix was used as input to construct a Monocle object. Genes showing significant inter-cluster variation were selected as ordering genes and used to model the progression of cellular states during differentiation. These genes were then applied to infer lineage trajectories and assign pseudotime values to individual cells. To evaluate the reliability of the inferred differentiation paths, differentially expressed genes were clustered based on their expression dynamics and visualized along the pseudotime axis.

### 2.8 Analysis of transcription factor-target gene regulatory network

To infer the core regulatory networks from single-cell RNA-seq data, we applied the SCENIC pipeline [22]. Briefly, the analysis began with the identification of co-expressed gene modules using GENIE3. Subsequently, TFs and their target genes were identified based on the mm10 RcisTarget database using RcisTarget software. Regulon activity scores were then calculated for each cell using AUCell with default settings. The entire SCENIC workflow was executed using the standard parameters provided by the SCENIC package (https://github.com/aertslab/SCENIC).

### 2.9 DNA methylation sequencing and analysis

To assess genome-wide DNA methylation patterns in granulosa cells, we employed the RRBS method. The granulosa cells were collected as input material for each sample. Genomic DNA from granulosa cells was extracted using the Oral Swab Genomic DNA Extraction Kit (Tiangen, DP322-03) according to the manufacturer's instructions. Bisulfite conversion of DNA was carried out using the Invitrogen MethylCode Bisulfite Conversion Kit, following the manufacturer's protocol. Following bisulfite treatment, DNA was amplified via two rounds of PCR amplification. PCR products were purified using Agencourt AMPure XP beads (0.8 × volume ratio) and size-selected (200–700 bp) using the QIAquick Gel Extraction Kit (Qiagen). Libraries were sequenced on the Illumina platform. Raw sequencing data were processed using Trim Galore to remove low-quality reads and adapter sequences, resulting in high-quality clean reads. Alignment to the mouse reference genome (mm10) and methylation calling were performed using Bismark software [23]. CpG sites with less than 5 × coverage were excluded from further analysis. DNA methylation levels across CpG sites were quantified and analyzed using the R package methylKit [24]. Pearson correlation coefficients between samples were calculated to assess data consistency, and hierarchical clustering and principal component analysis were performed to visualize methylation patterns across groups. DMRs were identified based on the following criteria: (1) at least five differentially methylated CpG sites located at corresponding genomic positions across samples; (2) coverage of ≥5 × ; and (3) a minimum 20% difference in average methylation level between groups. Genes associated with DMRs were subjected to GO enrichment analysis using the clusterProfiler R package.

### 2.10 Metabolites extraction

For metabolite extraction, tissue samples were weighed and an extraction solvent consisting of methanol:water (1:1) was added at a volume of 500 μL per 100 mg tissue. The samples were placed on ice and homogenized thoroughly. Following homogenization, the mixtures were vortexed for 30 s and incubated overnight at −20 °C to enhance metabolite solubilization and extraction efficiency. After incubation, the samples were vortexed again and centrifuged at 12,000×*g* for 12 min at 4 °C to pellet cellular debris. The resulting supernatants were transferred to clean microcentrifuge tubes and lyophilized under vacuum at low temperature to remove the solvent. The dried extracts were reconstituted in 100 μL of acetonitrile:water (1:1), vortexed for 30 s, and sonicated in an ice-water bath for 5 min to ensure complete resuspension. The samples were then centrifuged again at 12,000×*g* for 5 min at 4 °C to remove any insoluble material. An aliquot of the supernatant was transferred to a glass vial for subsequent LC-MS/MS analysis.

### 2.11 LC-MS/MS analysis

All LC-MS/MS analyses were performed using an ExionLC UPLC system (SCIEX, Framingham, MA) coupled to a TripleTOF® 5600 mass spectrometer (SCIEX). Chromatographic separation was achieved on a Waters HSS T3 column (100 × 2.1 mm, 1.8 μm) maintained at 40 °C. The mobile phases consisted of water containing 0.1% formic acid (phase A) and acetonitrile containing 0.1% formic acid (phase B). A linear gradient elution was applied with the flow rate set at 0.4 mL/min. The elution program was as follows: 0–1 min, 5% B; 1–8 min, 5–95% B; 8–10 min, 95% B; 10–12 min, 95–5% B; 12–14 min, 5% B. The autosampler was maintained at 4 °C, and the injection volume was set to 2 μL for both positive and negative ion modes. Mass spectrometric detection was carried out using an electrospray ionization (ESI) source in both positive and negative ionization modes. Data were acquired in Information-Dependent Acquisition (IDA) mode using Analyst® TF software (SCIEX). The acquisition parameters were as follows: primary MS scan range, 100–1200 *m*/*z*; accumulation time, 0.25 s; each MS scan triggered 12 dependent MS/MS scans (scan range: 50–1200 *m*/*z*; accumulation time: 0.05 s per scan). Dynamic background subtraction was enabled to improve spectral quality. The ion source parameters were optimized as follows: ion spray voltage, ±4500 V; source temperature, 550 °C; sheath gas, 30 psi; auxiliary gas (Gas1/Gas2), 55 psi. The collision energy was set to 40 eV with a ±20% fluctuation for both ionization modes.

### 2.12 Hematoxylin and Eosin (H&E) staining

Tissue sections were prepared from paraffin-embedded blocks by cutting 5 μm thick sections using a rotary microtome. Prior to staining, slides were deparaffinized in xylene followed by rehydration through graded ethanol solutions. Subsequently, sections were washed with phosphate-buffered saline (PBS). For H&E staining, sections were first stained with Harris hematoxylin solution (Sigma–Aldrich) followed by differentiation in 1% acid alcohol to remove excess stain and improve nuclear contrast. The sections were then briefly rinsed under running tap water for 1 min to develop the hematoxylin stain. Subsequently, sections were counterstained with eosin Y solution (Sigma–Aldrich) to visualize cytoplasmic structures. After staining, sections were dehydrated through an ascending series of ethanol concentrations, cleared in xylene, and mounted with coverslips.

### 2.13 Immunofluorescence analysis

For immunofluorescence staining, paraffin-embedded tissue sections (5 μm thick) were deparaffinized in xylene and rehydrated through a graded ethanol series into PBS. Antigen retrieval was performed by heating the sections in citrate buffer (pH 6.0) at 98 °C for 20 min, followed by cooling to room temperature. To block nonspecific binding and permeabilize cell membranes, sections were incubated in a blocking solution containing 5% bovine serum albumin (BSA) and 0.3% Triton X-100 in PBS for 1 h at room temperature. Sections were then incubated overnight at 4 °C with primary antibodies diluted in blocking buffer. The following primary antibodies were used: anti-Hsd3b1 (Santa Cruz, sc-515120, 1:200), anti-Cyp11a1 (Signalway, 32398, 1:200), anti-Lhcgr (Biorbyt, orb13542, 1:200). Following three washes with PBS, sections were incubated with species-appropriate fluorescently labeled secondary antibodies for 1 h at room temperature. Nuclei were counterstained with 4′,6-diamidino-2-phenylindole (DAPI; Life Technologies). Finally, sections were mounted under coverslips and examined using a confocal laser scanning microscope.

## 3 Results

### 3.1 Characterization and single cell transcriptome profiling of healthy and diabetic ovaries

**Figure 1:**
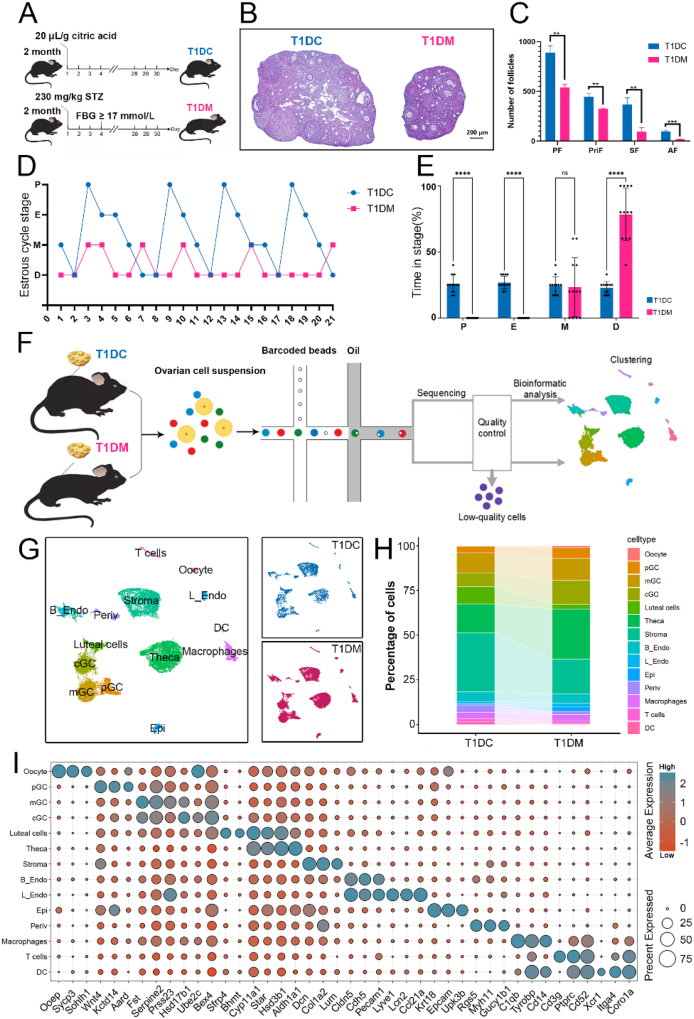
**Single-cell transcriptome profile of ovarian cells from T1DC and T1DM groups.** (A) Schematic diagram of the diabetic mouse model construction. (B) HE staining of ovaries in T1DC and T1DM groups. (C) The numbers of primordial follicles (PF), primary follicles (PriF), secondary follicles (SF) and antral follicles (AF) per ovary in T1DC and T1DM groups. (D) Representative estrous cycles from mice in the T1DC (blue) and T1DM (red) groups. P, proestrus; E, estrus; M, metestrus; D, diestrus. (E) Time distribution across estrous stages in T1DC (blue) and T1DM (red) mice. ∗∗∗∗P < 0.0001. (F) Experimental flowchart and analysis for scRNA-seq data, blue (or red) represents the T1DC (or T1DM) group. (G) A UMAP plot showing the annotated ovarian cell types (left). pGC, pre-antral granulosa cells; cGC, cumulus granulosa cells; mGC, mural granulosa cells; UMAP plot showing the distribution of cell types. Cells are colored by T1DC (blue) and T1DM (red) groups (right). (H) The percentage of ovarian cell types between T1DC and T1DM groups. (I) Dot plot showing the marker genes for different cell clusters, the expression level as shown on the color key at the right top. The percentage of cells expressing each gene was indicated by the size of the dot.

In this study, we sought to characterize the cellular and molecular alterations in ovaries induced by diabetes. To address this, we established a type 1 diabetic mouse model through streptozotocin administration (Figure 1A). Diabetic females exhibited significantly elevated fasting blood glucose levels, along with reduced body weight, smaller ovarian size, and decreased follicle count (Figure 1B,C; Figure S1A). Moreover, type 1 diabetic mice exhibit disrupted estrous cycles, characterized by prolonged time spent in diestrus (Figure 1D,E; Figure S1B). We then performed scRNA-seq on ovaries from both type 1 diabetes control (T1DC) and diabetic mice, using individual cell suspensions captured via droplet-based microfluidic device and subsequent sequencing (Figure 1F). Following stringent quality filtering and unbiased clustering analysis, we identified 14 distinct ovarian cell types, each exhibiting unique transcriptomic profiles (Figure 1G; Figure S1C,D).

Compared to controls, diabetic ovaries showed an increased proportion of theca cells and reduced proportions of luteal and stromal cells (Figure 1H). Cell clusters were annotated by comparing their gene expression patterns with known cell-type markers visualized in a dot plot (Figure 1I). We annotated oocytes, three types of granulosa cells (preantral follicular granulosa cells (pGC), mural granulosa cells (mGC), and cumulus granulosa cells (cGC)), luteal cells, theca cells, two types of endothelial cells (blood related endothelial cells (B_Endo) and lymphatic endothelial cells (L_Endo)), perivascular smooth muscle cells (Periv), epithelial cells (Epi) and stromal cells (Stroma) as well as three immune populations: T cells, dendritic cells (DC), and macrophages. We further analyzed differentially expressed genes (DEGs) across cell types and performed Gene Ontology (GO) enrichment analysis. The resulting biological processes were highly consistent with expected cellular identities, including responses to peptide hormones in theca cells, blood vessel development in B_Endo, and inflammatory responses in macrophages (Figure S1E). Together, these findings establish a comprehensive single-cell transcriptomic atlas of ovarian tissue in health and diabetes.

### 3.2 Global changes of the ovarian cells and their communications under diabetic condition

**Figure 2:**
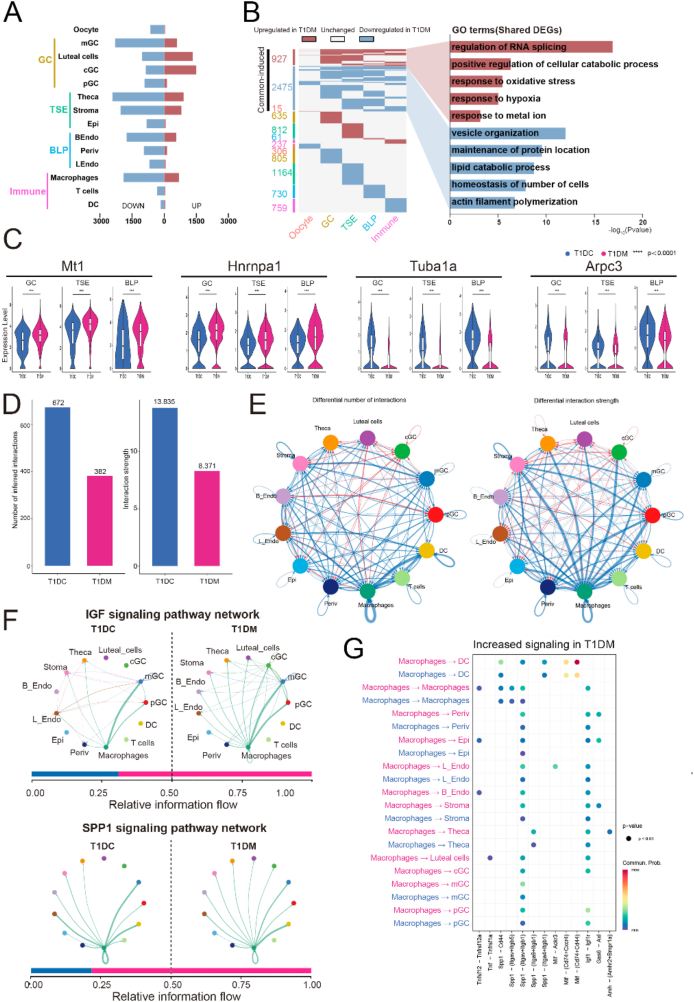
**Global alterations and cell communications of the ovarian cells in diabetes.** (A) Number of upregulated (red bar) and downregulated (blue bar) genes in each cell types. (B) Left: heatmap showing common and unique upregulated and downregulated DEGs betweenT1DC and T1DM groups in each major ovarian cell types. Right: representative GO terms of common-induced upregulated (top) or downregulated (bottom) DEGs between T1DC and T1DM groups and their associated p value. (C) Violin plots showing critical DEGs related to GO terms. (D) Bar plots showing the number of inferred interactions (left) or interaction strength (right) in the cell–cell communication network analyzed by CellChat. (E) Circle plots depict the differential number of interactions (left) or interaction strength (right) in the cell–cell communication network between T1DC and T1DM groups. (F) Circle plots showing selected inferred differential signaling networks. The edge width represents the communication probability. Bar graph at the bottom of each panel illustrates representative information flow in T1DC (blue) and T1DM (red). (G) Upregulated ligand–receptor signaling pairs between macrophages and other cell types.

To characterize diabetes-associated transcriptional changes in ovarian cells at the single-cell level, we performed cell type-specific differential gene expression analysis between T1DM and T1DC mice. Several cell types, including cGC, mGC, luteal cells, theca cells, stromal cells, B_Endo, and macrophages, exhibited the most pronounced alterations in gene expression under diabetic conditions (Figure 2A). Global analysis of DEGs across all cell types revealed a predominantly cell-type-specific response, with 1,760 upregulated and 3,764 downregulated genes. However, we also identified a subset of commonly dysregulated DEGs shared among two or more somatic cell types (upregulated: 927; downregulated: 2,475), suggesting conserved molecular responses to diabetes (Figure 2B). GO enrichment analysis of these commonly altered genes revealed that upregulated DEGs were enriched in biological processes such as “regulation of RNA splicing” and “response to oxidative stress,” whereas downregulated DEGs were primarily associated with “vesicle organization” and “actin filament polymerization” (Figure 2B). Representative genes included *Mt1*, a metallothionein involved in reactive oxygen species (ROS) detoxification [25]; *Hnrnpa1*, an RNA-binding protein linked to RNA splicing [26]; and *Tuba1a* and *Arpc3*, which are involved in cytoskeletal organization [27] (Figure 2C).

To further investigate cellular communication in the diabetic ovary, we applied CellChat to infer intercellular signaling interactions. Notably, both the number and strength of intercellular interactions were significantly reduced in the T1DM group compared to T1DC (Figure 2D). Macrophages, in particular, exhibited diminished communication with other cell types in diabetic ovaries, as reflected by reduced interaction numbers and strengths (Figure 2E, Figure S2A). For example, CCL signaling from macrophages to DC was markedly decreased (Figure S2B,C), as was VEGF signaling between macrophages and both B_Endo and L_Endo (Figure S2C). In contrast, several signaling pathways-including IGF, SPP1, and AMH-were enhanced between macrophages and other ovarian cell types in T1DM mice (Figure 2F, Figure S2D). To dissect the molecular basis of these changes, we systematically evaluated the expression of both ligands and their corresponding receptors across relevant cell populations. We found that *Igf1* was significantly upregulated in cGC under diabetic conditions, and its receptor *Igf1r* was concomitantly elevated in macrophages and theca cells (Figure S2E). Similarly, *Spp1* expression was markedly increased in macrophages, while its receptor *Itgav* was upregulated in cGC but downregulated in mGC. These findings suggest that the enhanced IGF and SPP1 signaling between cGC and macrophages likely results from coordinated upregulation of both ligands and receptors, whereas the increased SPP1 signaling between macrophages and mGC appears to be primarily driven by elevated ligand expression (Figure S2E). Moreover, quantitative CellChat analysis identified macrophages as a central signaling hub in the diabetic ovary, exhibiting increased communication probabilities with neighboring cell types via TNF, SPP1, IGF, and MIF pathways (Figure 2G). Taken together, these results indicate that diabetes profoundly alters intercellular communication within the ovarian microenvironment in a manner that may impair normal ovarian function.

### 3.3 Integrated analysis of gene expression and DNA methylation alterations in diabetic follicular cells

**Figure 3:**
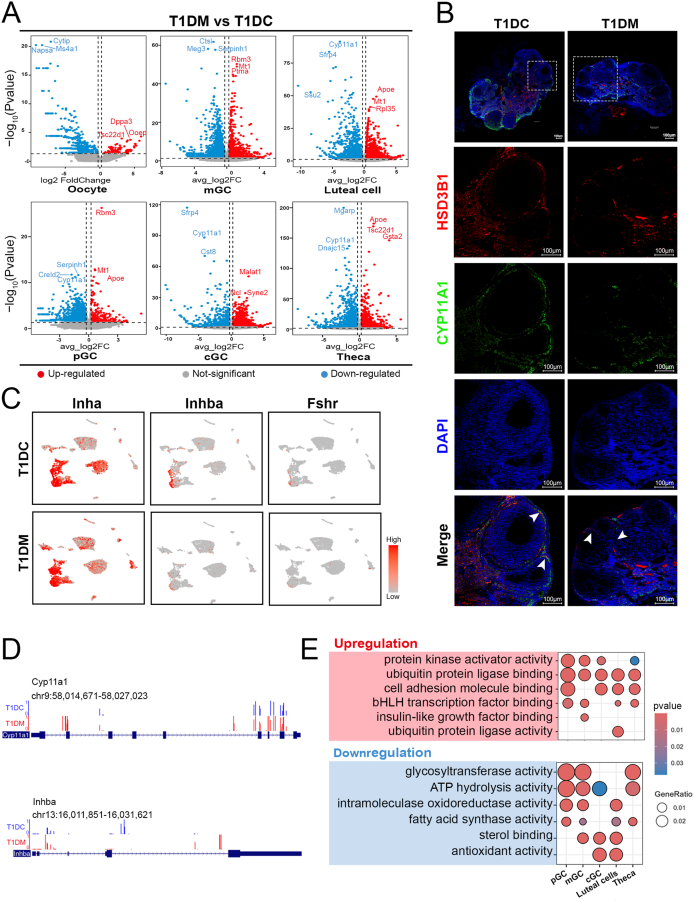
**Diabetes-related transcriptional and DNA methylation alterations in follicular cells.** (A) Volcano plot showing the significantly changed DEGs in the different follicular cell types in the T1DM compared with the T1DC groups. Red, upregulation; Blue, downregulation. (B) Immunostaining for Cyp11a1 and Hsd3b1 in ovary sections. DNA was counterstained with DAPI. Scale bar, 100 μm. (C) Expression patterns of key genes for folliculogenesis cast on the UMAP plot. (D) Methylation profile of the key genes in ovary. (E) Dot plot demonstrating gene ontology terms associated with DEGs in given clusters.

To comprehensively assess the molecular effects of diabetes on follicular cell populations, we analyzed DEGs in both oocytes and follicular somatic cells from T1DC and T1DM mice. Given that oocytes are typically excluded during microfluidic-based scRNA-seq due to their large size, we generated a dedicated oocyte transcriptomic library using Smart-seq2 [16], followed by DEG analysis using DESeq2 [28] (Figure 3A). For follicular somatic cells, DEGs were identified using the FindMarkers function in the Seurat package [17] (Figure 3A). We observed that *Cyp11a1*, a key gene involved in steroid hormone synthesis [29], was significantly downregulated across most follicular cell types in diabetic ovaries, whereas *Mt1 (Metallothionein 1)*, an antioxidant gene, was upregulated in pGC, mGC, and luteal cells (Figure 3A,B). Further investigation of critical regulators of folliculogenesis revealed that *Inha* was markedly reduced in theca cells, while *Inhba* and *Fshr* were significantly downregulated in both cGC and mGC in the T1DM group (Figure 3C, Figure S3A).

Given the potential role of epigenetic regulation in diabetes-induced ovarian dysfunction, we next examined DNA methylation changes in granulosa cells using reduced representation bisulfite sequencing (RRBS) (Figure S3B). Analysis of differentially methylated regions (DMRs) revealed distinct patterns associated with gene regulatory functions. GO enrichment of DMR-associated genes showed that hypermethylated regions were enriched in biological processes such as “cell junction assembly” and “response to transforming growth factor beta,” while hypomethylated regions were linked to pathways including “Notch signaling” and “response to hypoxia” (Figure S3C). Notably, promoter methylation levels of representative genes were altered in diabetic granulosa cells: *Cyp11a1* and *Inhba* exhibited increased methylation (Figure 3D).

To further explore the functional implications of transcriptional dysregulation in follicular cells, we performed GO enrichment analysis on DEGs across all follicular subtypes. Upregulated genes were predominantly associated with “protein kinase activator activity” and “ubiquitin protein ligase binding”, while downregulated genes were enriched in “ATP hydrolysis activity, intramolecular” and “sterol binding” (Figure 3E). Importantly, we also identified cell-type-specific functional alterations by analyzing unique GO terms per subtype. These included reduced “glucosidase activity” in pGC, “lipid phosphatase activity” in mGC, “sterol transporter activity” in cGC, “CoA hydrolase activity” in luteal cells, and “adenyl transferase activity” in theca cells (Figure S3D). Collectively, these findings demonstrate that T1DM induces extensive, cell-type-specific disruptions in steroidogenic and metabolic pathways, which may contribute to impaired ovarian function.

### 3.4 Disrupted granulosa cell differentiation in diabetes is associated with altered gene expression and DNA methylation patterns

**Figure 4:**
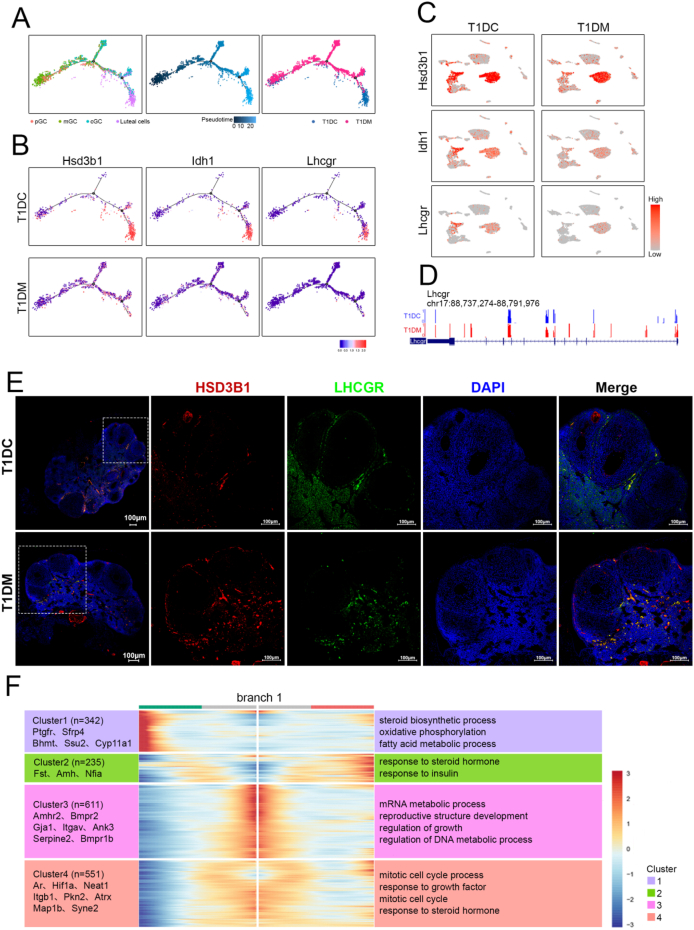
**Gene expression and DNA methylation alterations in granulosa cells under diabetic conditions.** (A) Pseudotime trajectory of granulosa and luteal cells analyzed by Monocle. (B) Expression of critical DEGs along pseudotime trajectory. (C) Expression patterns of critical DEGs cast on the UMAP plot. (D) Methylation profile of the critical gene in ovary. (E) Immunostaining for Hsd3b1 and Lhcgr in ovary sections. DNA was counterstained with DAPI. Scale bar, 100 μm. (F) Pseudotemporal expression pattern analysis shows the representative DEGs in 4 clusters, along with the inferred trajectory and enriched biological processes terms.

Given the observed disruptions in follicular development, we next aimed to characterize the cellular and molecular mechanisms underlying impaired granulosa cell differentiation in the T1DM group. To achieve this, we constructed pseudotime trajectories using Monocle2 for granulosa and luteal cells from both T1DC and T1DM mice (Figure 4A). Striking differences were evident between the inferred developmental trajectories: granulosa cells from diabetic mice exhibited an incomplete differentiation process and failed to fully transition into luteal cells (Figure 4A). Notably, luteal cells were predominantly localized at the terminal stage of the trajectory in control mice, suggesting that diabetes disrupts the normal progression of granulosa-to-luteal cell differentiation (Figure 4A). We further analyzed gene expression dynamics along the pseudotime trajectories and identified several key genes whose expression was significantly altered in diabetic granulosa cells. Among these, *Hsd3b1*, *Idh1*, and *Lhcgr*, which are key regulators of hormone biosynthesis and signaling pathways, were markedly downregulated in the T1DM group (Figure 4B). Examination of their expression patterns across both groups confirmed a significant reduction in granulosa and luteal cells from diabetic mice, with *Lhcgr* showing the most pronounced decrease (Figure 4C). This downregulation suggests a diminished capacity of granulosa cells to respond to LH, potentially impairing luteal cell formation.

To validate the transcriptional downregulation of *Lhcgr*, we examined its DNA methylation profile in granulosa cells. Our analysis revealed increased methylation levels at the *Lhcgr* locus in the diabetic group, indicating a potential epigenetic mechanism contributing to its reduced expression (Figure 4D). Consistently, immunofluorescence staining showed a marked decrease in Lhcgr protein levels in ovarian tissues from T1DM mice, corroborating our transcriptomic findings (Figure 4E). To further elucidate the dynamic changes in granulosa cell states during differentiation, we performed pseudotime-dependent DEGs profiling and GO enrichment analysis across trajectory clusters (Figure 4F). Cluster 1 consisted of genes such as *Sfrp4*, *Ptgfr*, and *Cyp11a1*, which were highly expressed at the terminal stage and enriched in biological processes related to steroid biosynthesis and fatty acid metabolism. Clusters 2 and 3 contained genes like *Amhr2* and *Fst*, which were predominantly expressed at the early stages of the trajectory and are known to be involved in reproductive organ development and steroid hormone signaling pathways. Cluster 4 included genes such as *Ar*, *Hif1a*, and *Itgb1*, which showed peak expression in the middle stages and were associated with mitotic cell cycle regulation and responses to growth factors. Taken together, these results demonstrate that diabetes profoundly disrupts the differentiation trajectory and functional maturation of ovarian granulosa cells, with implications for impaired folliculogenesis and luteinization.

### 3.5 Altered regulatory networks and metabolic profiles in granulosa and luteal cells under diabetic conditions

**Figure 5:**
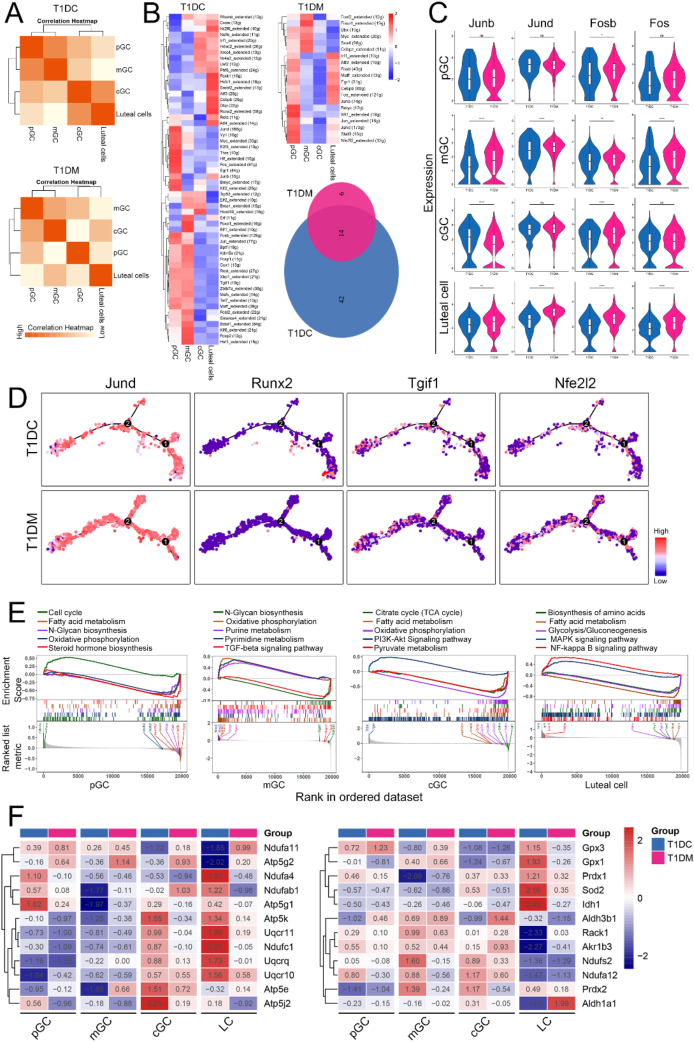
**Changes in regulatory transcription factors and metabolic profiles in granulosa and luteal cells during diabetes.** (A) Heatmap showing the correlations among different cell clusters. (B) Heatmap of regulon activity in T1DC and T1DM groups analyzed by SCENIC. Venn diagram of differentially expressed transcription factors in T1DC and T1DM groups (right bottom). (C) Violin plots showing shared transcription factors in T1DC and T1DM groups. (D) Expression of critical transcription factors along pseudotime trajectory. (E) GSEA analysis of gene sets in granulosa and luteal cells. (F) Heatmap showing the expression levels of genes involved in oxidative phosphorylation (left) and redox homeostasis (right).

Following ovulation, granulosa cells undergo terminal differentiation into luteal cells. To assess how diabetes affects the cellular relationships between granulosa and luteal cell populations, we performed correlation analysis across T1DC and T1DM samples. In T1DC group, pGC clustered closely with mGC. In contrast, in T1DM group, pGC showed higher similarity to luteal cells than to other granulosa cell subtypes (Figure 5A). To further characterize the transcriptional regulatory changes associated with diabetes, we applied single-cell regulatory network inference and clustering (SCENIC) to infer key transcription factor (TF) networks in granulosa and luteal cells. Our analysis revealed a marked reduction in the number of enriched TFs in the T1DM group compared to controls (Figure 5B). Notably, the similarity in TF enrichment profiles between pGC and luteal cells was significantly increased under diabetic conditions, suggesting a partial convergence of their transcriptional programs. A venn diagram identified 14 common TFs shared between T1DC and T1DM groups (Figure 5B). Based on their potential regulatory roles, we selected Junb, Jund, Fosb, and Fos for detailed expression analysis. These TFs were generally upregulated in most ovarian cell types in the diabetic group. However, Junb exhibited an inverse pattern, showing significant downregulation specifically in cGC under diabetic conditions (Figure 5C). To further explore the dynamics of transcriptional regulation during granulosa to luteal cell differentiation, we mapped the expression of key transcription factors onto pseudotime trajectories (Figure 5D). We found that Jund exhibited relatively uniform expression throughout the trajectory, suggesting a constitutive regulatory role. In contrast, Runx2 and Tgif1 were markedly downregulated specifically along branch 1 of the pseudotime trajectory in the T1DM group. Conversely, Nfe2l2 showed significantly elevated expression across the same branch, implying a potential compensatory or stress responsive transcriptional shift in diabetes (Figure 5D).

To explore the functional consequences of these transcriptional alterations, we conducted gene set enrichment analysis (GSEA) across granulosa and luteal cell populations. Several metabolic pathways were significantly affected in diabetic cells. The fatty acid metabolism pathway was broadly suppressed in pGC, cGC, and luteal cells. Additionally, N-glycan biosynthesis was reduced in pGC and mGC, pyruvate metabolism was downregulated in cGC, and glycolysis was decreased in luteal cells (Figure 5E). Interestingly, oxidative phosphorylation was significantly decreased in pGC and cGC but increased in mGC under diabetic conditions (Figure 5E). Further analysis of oxidative phosphorylation-related genes confirmed this trend: overall expression levels were reduced in pGC, cGC, and luteal cells from T1DM mice, while mGC showed elevated expression (Figure 5F). Given the observed disruption in mitochondrial function, we examined the expression of key antioxidant genes involved in redox homeostasis. Diabetic luteal cells showed significant downregulation of *Gpx1*, *Gpx3*, *Prdx1*, *Prdx2*, *Idh1*, and *Sod2*, reflecting a diminished ability to counteract oxidative stress and maintain redox equilibrium. In contrast, *Aldh1a1* was markedly upregulated in luteal cells in the T1DM group (Figure 5F). This distinct expression pattern suggests a possible compensatory response to increased oxidative stress, potentially mediated through *Aldh1a1*-dependent detoxification mechanisms.

### 3.6 Effects of diabetes on ovarian metabolome

**Figure 6:**
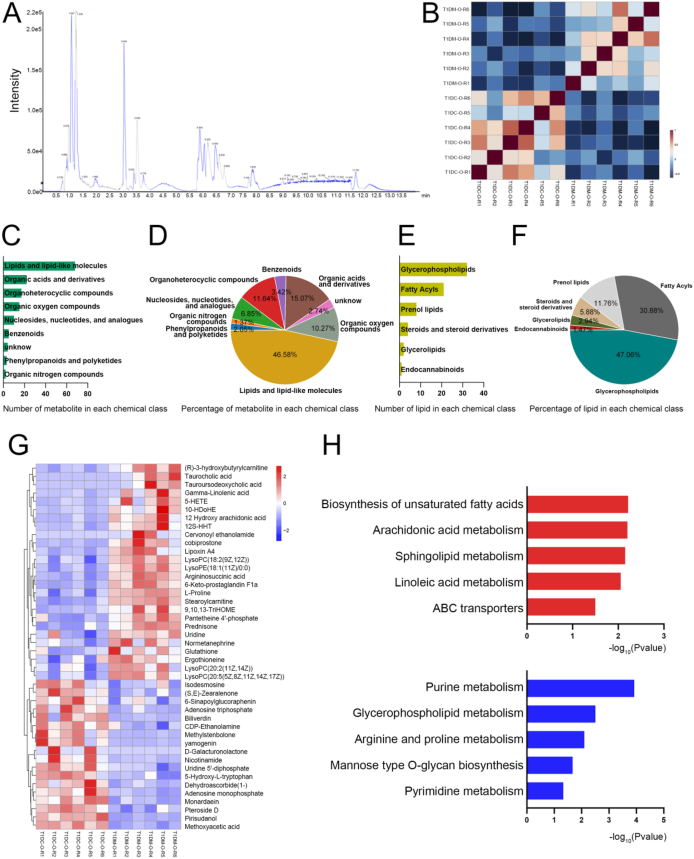
**Overview of the mouse ovary metabolome dataset.** (A) Base peak chromatogram showing the most intense ion detected at each retention time. (B) Heatmap illustrates the correlation among replicate samples within each group. (C) Number of the detected metabolites in each chemical class using HMDB classification scheme. (D) Percentage of each chemical class in the metabolites. (E) Number of the detected lipids in each chemical class. (F) Percentage of each lipid class. (G) Heatmap of the differentially expressed metabolites in different conditions. (H) Pathway analysis of the highly expressed and lowly expressed metabolites.

To characterize the global metabolic alterations induced by diabetes in the ovary, we performed LC-MS/MS-based metabolomic profiling of ovarian tissues from STZ-treated and control C57BL/6 female mice (Figure 6A). Sample correlation analysis within each experimental group demonstrated strong inter-replicate consistency, confirming high reproducibility and data reliability (Figure 6B). In total, 3,422 metabolic features were detected in negative ionization mode, and 3,160 features were identified in positive ionization mode (Supplementary Data 1). To identify differentially expressed metabolites (DEMs) between the T1DC and T1DM groups, we conducted orthogonal partial least-squares discrimination (OPLSD) analysis, which revealed 210 statistically significant DEMs. The distribution and proportion of 146 DEMs with HMDB ID across various chemical categories are summarized in Figure 6C,D. After filtering out lipid species detected redundantly by both ionization modes, as well as those with low signal-to-noise ratios or high coefficients of variation, we retained 68 unique lipid species representing 6 distinct lipid classes for downstream analysis (Figures 6E,F). Hierarchical clustering of the most significantly altered metabolites showed that several metabolites, including LysoPC, 5-HETE, argininosuccinic acid, and stearoylcarnitine, were elevated in the T1DM group. In contrast, isodesmosine, monardaein, and pteroside D were markedly reduced in diabetic ovaries (Figure 6G). To further interpret these metabolic changes in a biological context, we performed pathway enrichment analysis using the identified DEMs (Figure 6H). Notably, upregulated metabolites were predominantly associated with pathways related to biosynthesis of unsaturated fatty acids and sphingolipid metabolism, whereas downregulated metabolites were enriched in purine metabolism and glycerophospholipid metabolism (Figure 6H). Collectively, these results reveal a profound metabolic reprogramming in the diabetic ovary, highlighting dysregulation in lipid and energy metabolism pathways that may contribute to ovarian dysfunction in the context of type 1 diabetes.

## 4 Discussion

The impact of diabetes on ovarian function is complex and multifactorial, yet the underlying molecular mechanisms remain incompletely characterized. A comprehensive understanding of the epigenetic, transcriptional, and metabolic perturbations in the diabetic ovary is essential for elucidating how metabolic dysregulation compromises reproductive physiology. In this study, we employed a multi-omics approach integrating single-cell RNA sequencing, DNA methylation profiling, and untargeted metabolomics to construct a comprehensive atlas of the ovarian response to type 1 diabetes in a mouse model. Our analyses revealed profound alterations in key signaling pathways (Figure 2D–F), granulosa cell differentiation trajectories (Figure 4), and lipid metabolism (Figure 6), highlighting the interconnected nature of these disruptions in the diabetic ovary. Collectively, these findings provide novel mechanistic insights into diabetes-associated ovarian dysfunction and establish a valuable resource for future investigations into metabolic reproductive disorders.

Remarkable differences on ovarian transcriptional landscape were observed under diabetic conditions, with notable changes in the expression of key regulators of redox homeostasis, lipid metabolism, and steroidogenesis. Our single-cell transcriptomic analysis revealed upregulation of *Mt1* and *Apoe* (Apolipoprotein E), along with downregulation of *Cyp11a1* and *Fshr*, in follicular cells (Figure 3A). *Mt1* is a small, cysteine-rich metal-binding protein with well-established roles in metal ion homeostasis and ROS scavenging [30]. The upregulation of *Mt1* in diabetic ovaries suggests a compensatory response to counteract increased oxidative stress, which is a hallmark of diabetes-associated tissue damage. Consistent with previous studies showing that *Mt1* overexpression preserves angiogenic signaling in diabetic models through stabilization of the HIF-1α/SDF-1/VEGF pathway [31,32], our findings support a protective role for *Mt1* in the diabetic ovary. Similarly, the increased expression of *Apoe*, a key component of lipoprotein particles with anti-inflammatory and antioxidant properties, was predominantly observed in luteal and theca cells. Given its role in lipid transport and immune modulation [33], the upregulation of *Apoe* may represent an adaptive mechanism aimed at preserving cellular integrity and steroidogenic capacity under diabetic conditions. In contrast, the downregulation of *Cyp11a1* and *Fshr* in granulosa cells indicates a major disruption in the molecular machinery governing steroid hormone biosynthesis and follicular development. *Cyp11a1* encodes the rate-limiting enzyme in steroidogenesis, responsible for the conversion of cholesterol to pregnenolone-the precursor for all major steroid hormones, including estrogen and progesterone [34]. Reduced *Cyp11a1* expression likely impairs steroidogenic output, potentially leading to hormonal imbalances that compromise follicular maturation and ovulatory function [35]. Moreover, the decreased expression of *Fshr*, which encodes the follicle-stimulating hormone receptor, suggests a diminished capacity of granulosa cells to respond to gonadotropic stimulation. Given the essential role of FSHR signaling in promoting early follicular growth and preventing atresia through the FSHR-mTOR-HIF1 pathway [36], this downregulation may contribute to impaired folliculogenesis and subfertility in diabetic females.

Ovarian follicle development and ovulation are tightly regulated by complex intercellular signaling networks. The integration of single-cell transcriptomic data enabled us to dissect the cellular communication landscape within the ovary and revealed significant alterations in signaling pathways under diabetic conditions. Our analysis identified a widespread reduction in intercellular interactions in the T1DM group (Figure 2D), suggesting a general disruption in ovarian cell coordination. However, certain signaling pathways exhibited compensatory activation, most notably the IGF signaling pathway, which was significantly upregulated in diabetic ovaries (Figure 2F). The IGF signaling pathway plays a critical role in mammalian fertility by promoting follicular growth, enhancing steroid hormone production, and supporting granulosa cell survival [37]. Although circulating IGF1 levels are known to decline following the onset of type 1 diabetes [38], our findings suggest that local activation of the IGF pathway within the ovary may represent an adaptive response to counteract diabetes-induced cellular stress or dysfunction. Similarly, in patients with T1DM undergoing insulin administration, higher circulating insulin levels can bind to IGF receptors on ovarian theca, granulosa, and stromal cells, thereby stimulating androgen secretion and contributing to the development of polycystic ovary syndrome (PCOS)-like features [39,40]. In contrast, we observed a significant downregulation of the VEGF signaling axis between luteal cells, stromal cells and macrophages and ovarian endothelial cells in the T1DM group (Figure S2C). Consistent with our findings, studies in mouse models of T2DM have demonstrated that diabetes induces ovarian dysfunction through the suppression of ovarian angiogenesis, mediated by downregulation of the VEGF signaling pathway [41]. Although ovarian VEGF signaling in women with T1DM is poorly characterized, systemic suppression of VEGF in T1DM suggests impaired ovarian angiogenesis and endothelial function, potentially contributing to ovarian dysfunction [42]. Additionally, AMH, primarily secreted by granulosa cells of preantral and early antral follicles, functions as a key regulator of follicle recruitment and growth by inhibiting the activation of primordial follicles [43,44]. Disruption of AMH signaling may therefore contribute to altered follicular dynamics and potentially accelerate ovarian reserve depletion in diabetic conditions. These findings highlight the involvement of AMH signaling in diabetes-associated ovarian dysfunction and suggest that its dysregulation warrants further investigation as a potential therapeutic target.

Our pseudotime trajectory analysis revealed a significant impairment in granulosa cell differentiation in diabetic ovaries. Specifically, we observed an incomplete transition toward the luteal cell lineage and a reduced abundance of fully mature luteal cells. This transformation is a key step in the formation of the corpus luteum and is essential for maintaining normal ovulatory and hormonal function. At the molecular level, we identified significant downregulation of several genes critical for luteal differentiation and hormonal responsiveness, including *Hsd3b1*, *Idh1*, and *Lhcgr*. Among these, the reduced expression of *Lhcgr* is of particular importance. *Lhcgr* encodes the luteinizing hormone receptor, which plays a central role in mediating the effects of the preovulatory LH surge and initiating the luteinization of granulosa cells [37,45,46]. The observed downregulation of *Lhcgr* suggests a diminished capacity of granulosa cells to respond to LH stimulation, which may compromise the granulosa-to-luteal cell transition and ultimately lead to defective corpus luteum formation. Our multi-omics analysis further supports this hypothesis by revealing increased DNA methylation at the *Lhcgr* locus in diabetic ovaries, along with reduced Lhcgr protein expression. These findings suggest that epigenetic mechanisms may contribute to the transcriptional suppression of *Lhcgr*, providing a potential link between metabolic stress and impaired LH signaling in the diabetic ovary [47]. This observation not only highlights the role of epigenetic regulation in diabetes-associated ovarian dysfunction but also offers new insights into the molecular basis of reproductive impairments under metabolic disease conditions.

Metabolomic profiling of ovarian tissues from our type 1 diabetic mouse model revealed extensive metabolic reprogramming, reflecting the profound impact of systemic metabolic dysregulation on ovarian physiology. For example, in this study, we observed a significant downregulation of oxidative phosphorylation in multiple subtypes of granulosa cells, revealing a pattern of mitochondrial dysfunction that parallels the impairment of this pathway previously documented in skeletal muscle from human patients with type 2 diabetes [48]. Using LC-MS/MS-based metabolomics, we identified a large number of DEMs, with pathway enrichment analysis highlighting significant disturbances in lipid and energy metabolism. Among the upregulated metabolites, elevated levels of LysoPC and 5-HETE suggest increased lipid peroxidation and activation of inflammatory signaling pathways, which may contribute to oxidative stress and disrupt follicular development [49,50]. Similarly, patients with T1DM display elevated rates of myocardial fatty acid oxidation, as previously demonstrated in clinical metabolic studies [51]. Additionally, increased stearoylcarnitine levels point to impaired mitochondrial fatty acid oxidation, potentially compromising cellular energy homeostasis in ovarian cells [52]. Conversely, the significant depletion of metabolites such as glycerophospholipids, isodesmosine and pteroside D indicates a reduction in antioxidant effect, anti-inflammatory and protective molecules [[53], [54], [55]]. Interestingly, recent findings suggest that optimal glycemic control in individuals with new-onset T1DM is associated with potentially beneficial alterations in glycerophospholipid metabolism. Further investigation is warranted to elucidate the role of these metabolic shifts in the pathogenesis of diabetes-related ovarian dysfunction [56]. Moreover, the glycerophospholipid metabolism pathway is significantly downregulated in patients with diminished ovarian reserve and advanced age [57]. This metabolic shift further supports the notion of a pro-oxidative and metabolically compromised ovarian microenvironment in diabetes. Collectively, these metabolic alterations highlight the central role of metabolic dysregulation in the pathogenesis of ovarian dysfunction in type 1 diabetes. The identification of distinct metabolic signatures in diabetic ovaries not only deepens our understanding of the molecular mechanisms linking metabolic stress and reproductive dysfunction but also provides a foundation for the discovery of novel biomarkers and potential therapeutic strategies targeting metabolic pathways in metabolic-reproductive disorders.

In conclusion, our multi-omics profiling of the ovarian landscape in a diabetic mouse model reveals comprehensive molecular alterations associated with diabetes exposure, offering a valuable resource of potential therapeutic targets and biomarkers for assessing the impact of metabolic dysfunction on ovarian health. By leveraging single-cell resolution, this study provides novel insights into the cellular and molecular mechanisms by which diabetes may impair ovarian function, laying a foundation for future investigations into the interplay between metabolic and reproductive systems.

## CRediT authorship contribution statement

**Zheng-Hui Zhao:** Writing – original draft, Validation, Funding acquisition, Formal analysis. **Xue-Ying Chen:** Visualization, Validation, Software. **Cheng-Yan Zhuo:** Methodology. **Xiang-Hong Ou:** Supervision, Resources, Methodology. **Qing-Yuan Sun:** Writing – review & editing, Supervision, Funding acquisition, Conceptualization.

## Funding

This work was supported by National R&D program of China, Grant/Award Number: 2022YFC2703501; Guangdong Basic and Applied Research Foundation, Grant/Award Number: 2023B1515120027; National Natural Science Foundation of China, Grant/Award Number: 82401895, 82530050.

## Declaration of competing interest

The authors declare no competing interests.

## Data Availability

Data will be made available on request.
