# Supplementary material for: Multi-omics atlas of ovarian cellular and molecular responses to diabetes
Source: Mol Metab. 2025 Dec 13;103:102307. doi: 10.1016/j.molmet.2025.102307 (PMC12808612; doi:10.1016/j.molmet.2025.102307)
Supplement: Multimedia component 2 [file mmc2.docx]

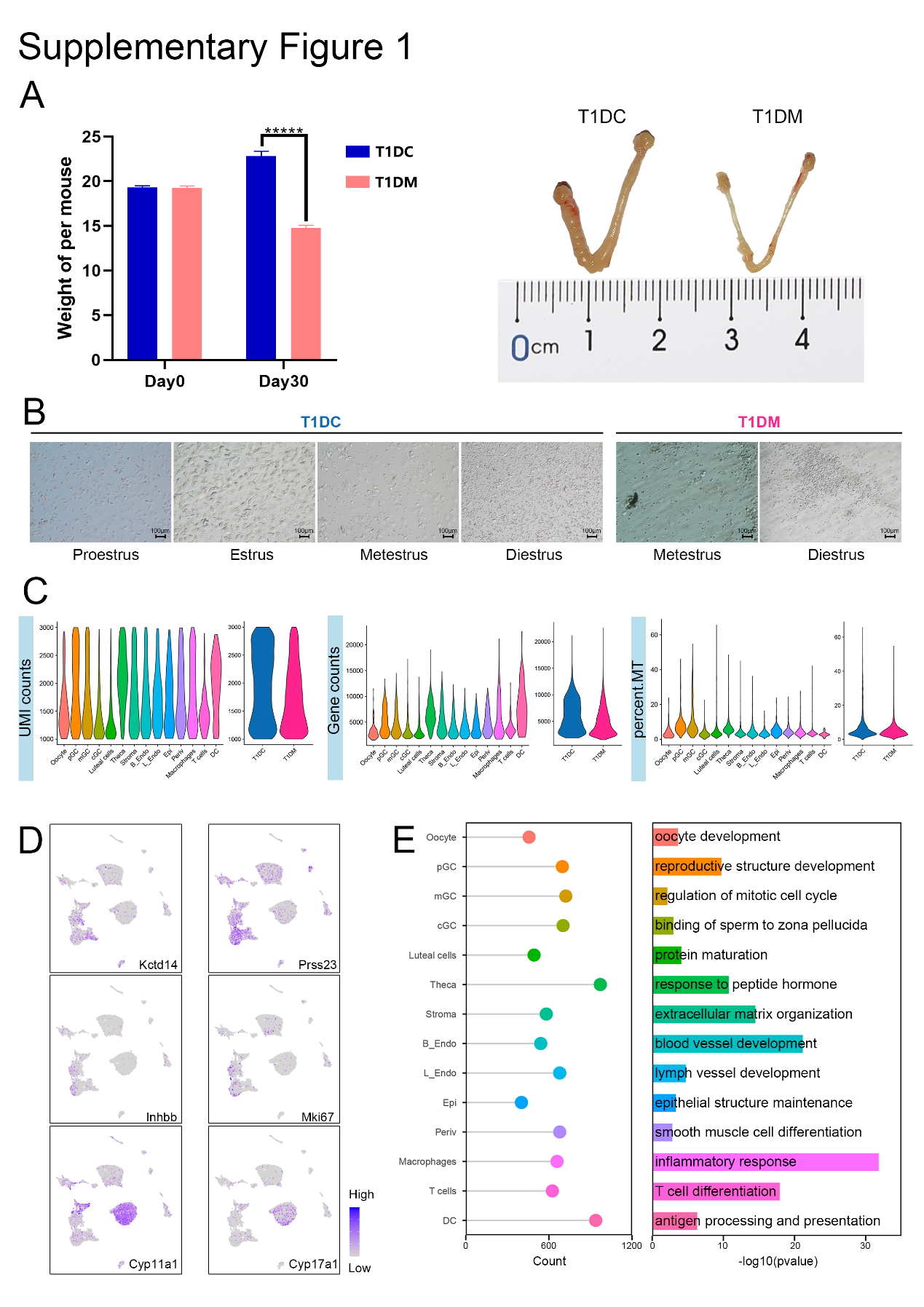


**Figure S1 Overview of single cell transcriptional atlas.** (A) Changes in body weight and ovarian as well as uterine morphology under diabetic conditions. (B) Estrous stage classification based on vaginal cytology in T1DC and T1DM mice. (C) Violin plot showing the UMI counts, gene counts and percentage of mitochondrial genes in each cell type and group. (D) Expression pattern of selected markers cast on the UMAP plot. (E) Lollipop chart illustrates the numbers of cell marker genes (left panel), and bar plot represents the cell type-specific enriched biological pathways.


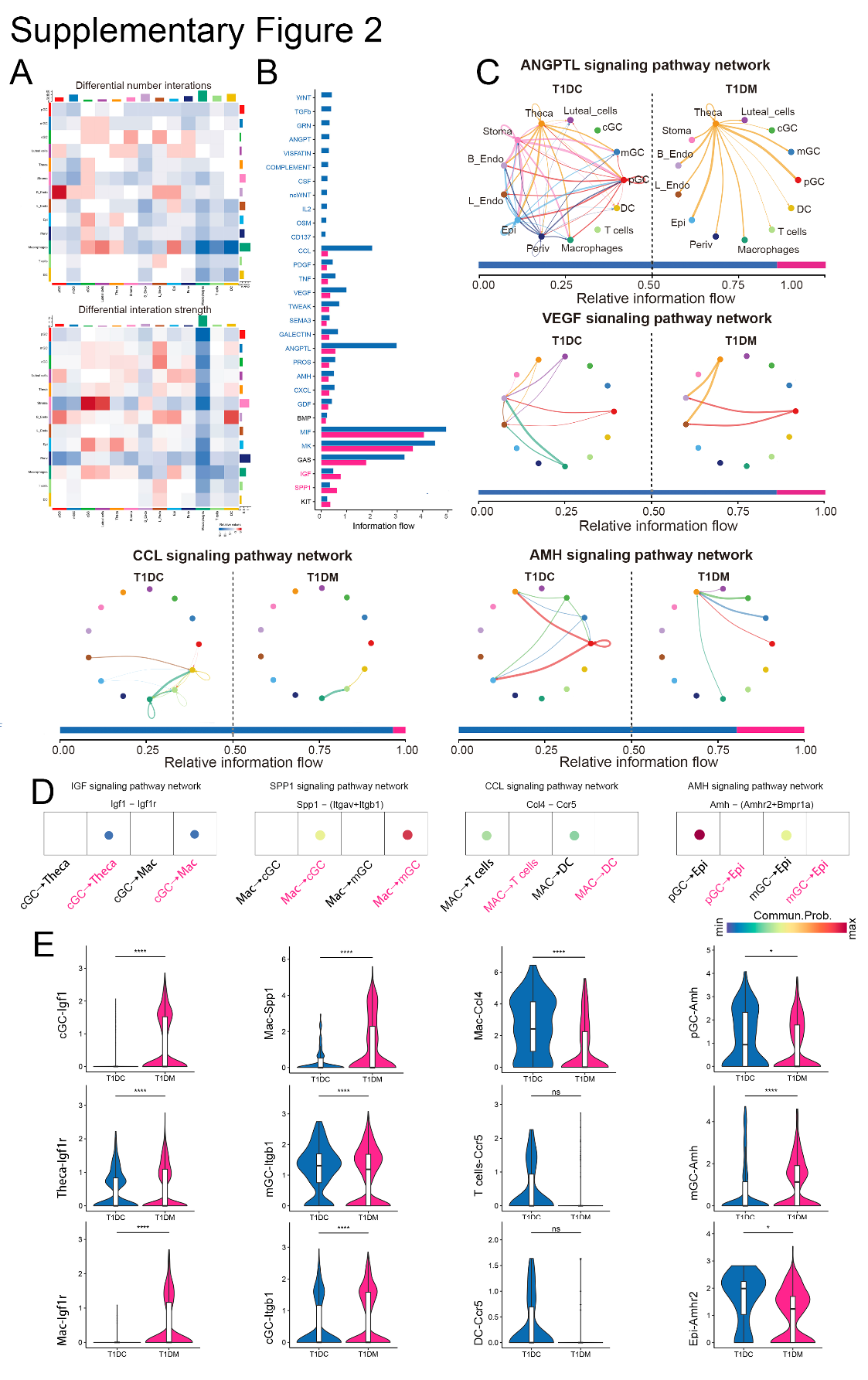


**Figure S2 Global cell-cell interactions in diabetic ovaries.** (A) Heatmaps show the differential interaction numbers and strength between T1DM vs. T1DC groups. (B) The representative information flow for typical signaling pathways in T1DC (blue) and T1DM (red). (C) Circle plots showing selected inferred differential signaling networks. The edge width represents the communication probability. (D) Bubble plot indicates the differential signaling pathways between T1DC and T1DM groups. (E) Violin plots showing the expression levels of ligands and receptors between T1DC and T1DM groups.


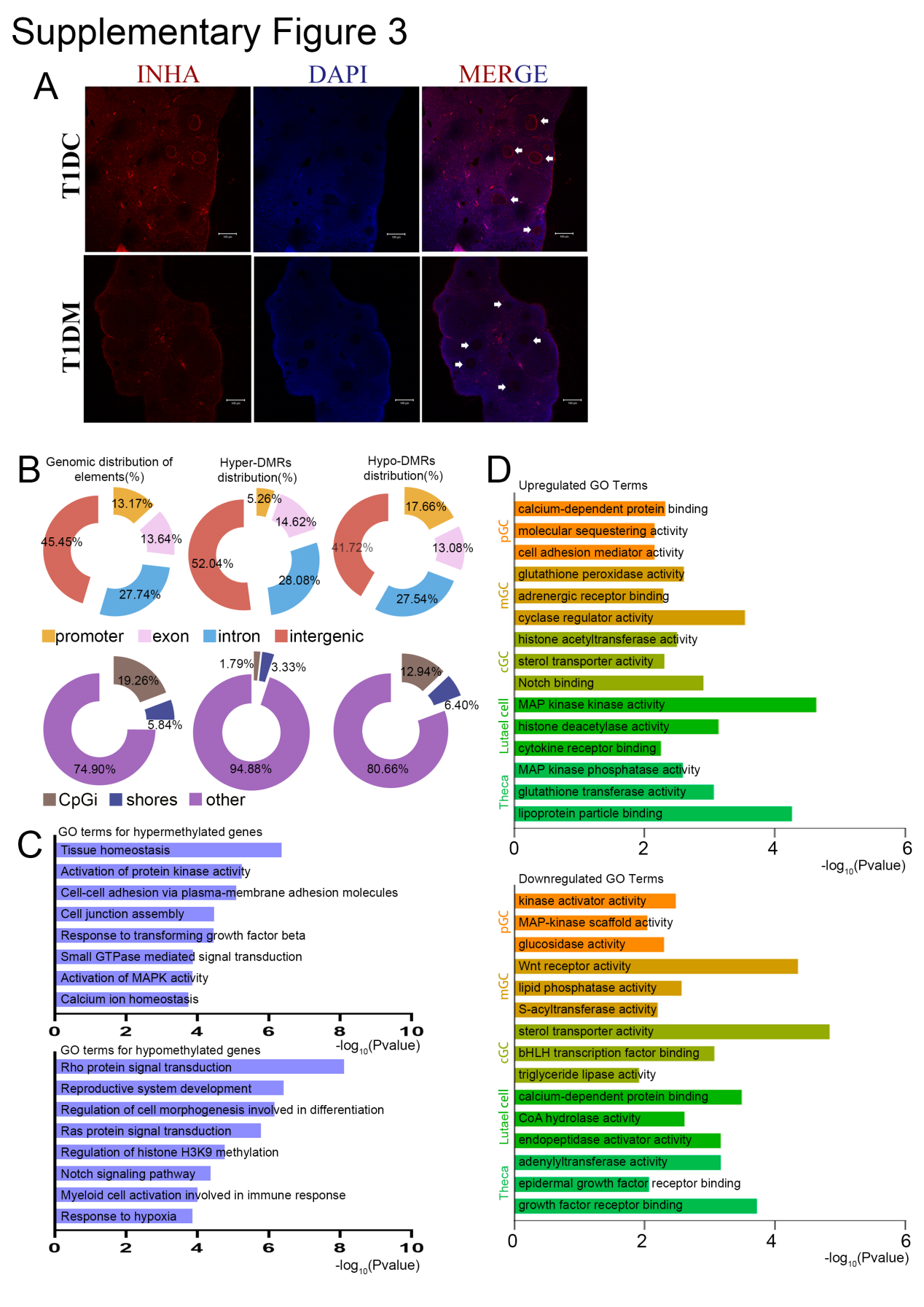


**Figure S3 Altered DNA methylation profiles and biological processes.** (A) Immunostaining for Inha in ovary sections. DNA was counterstained with DAPI. Scale bar, 100 µm. (B) The relative proportion of DMRs across the genome. (C) The GO terms for DMRs associated genes. (D) Unique GO terms for subtypes of granulosa cells and luteal cells.
